# Knee complaints and consequences on work status; a 10-year follow-up survey among floor layers and graphic designers

**DOI:** 10.1186/1471-2474-8-93

**Published:** 2007-09-18

**Authors:** Søren Rytter, Lilli Kirkeskov Jensen, Jens Peter Bonde

**Affiliations:** 1 Department of Occupational Medicine and Orthopaedics, Region Hospital Skive, Viborg, Denmark; 2 Department of Occupational Medicine, Region Hospital Skive, Denmark; 3 Department of Occupational Medicine, Aarhus University Hospital, Aarhus, Denmark

## Abstract

**Background:**

The purpose of the study was to examine if knee complaints among floor layers predict exclusion from the trade.

**Methods:**

In 1994/95 self-reported data were obtained from a cohort of floor layers and graphic designers with and without knee straining work activities, respectively. At follow-up in 2005 the questionnaire survey was repeated. The study population consisted of 81 floor layers and 173 graphic designers who were presently working in their trades at baseline (1995). All participants were men aged 36–70 years in 2005.

We computed the risk of losing gainful employment in the trade according to occurrence of knee complaints at baseline, using Cox proportional hazard regression adjusted for a number of potential confounding variables. Moreover, the crude and adjusted odds risk ratio for knee complaints according to status of employment in the trade were computed, using graphic designers as reference.

**Results:**

A positive but non-significant association between knee complaints lasting more than 30 days the past 12 months and exclusion from the trade was found among floor layers (Hazard Ratio = 1.4, 95% CI = 0.6–3.5).

The frequency of self-reported knee complaints was lower among floor layers presently at work in the trade in year 2005 (26.3%) compared with baseline in 1995 (41.1%), while the opposite tendency was seen among graphic designers (20.7% vs. 10.7%).

**Conclusion:**

The study suggests that knee complaints are a risk factor for premature exclusion from a knee demanding trade. However, low power of the study precludes strong conclusions. The study also indicates a healthy worker effect among floor layers and a survivor effect among graphic designers.

## Background

Musculoskeletal disorders are very common in the general population and are the predominant cause of disability among construction workers [1]. Previous studies have shown an increased frequency of self-reported and clinically diagnosed knee disorders and radiological diagnosed knee osteoarthritis among workers within certain trades in the construction industry [2-8].

There are about 900 skilled floor layers in Denmark and they spend on average more than half of their daily working time in knelling, knee supporting or squatting work positions. Floor layers are highly exposed to various knee-stressing work positions in their work tasks, defined as work in kneeling positions, completely on both or partly on one knee using the other as support, and as squatting work positions. They install linoleum, carpet and vinyl floorings and their work tasks involve removal of old floorings, grinding, filling, installing underlay, measuring and cutting materials, gluing, welding and installing skirting board (plastic). Graphic designers handle the layout of text and use visual display units. Their work tasks did not include any knee-demanding work positions.

Compared with other skilled construction workers, floor layers have a high frequency of musculoskeletal complaints, particularly anterior knee pain. This has been observed among Danish, as well as American, Finnish and Swedish floor layers [8-12]. Two earlier studies have shown that floor layers have an increased risk of prolonged sick leave and premature retirement from the trade [9,13]. This might be due to the high prevalence of knee disorders in this trade.

Therefore the primary aim of the current study was to analyse whether knee complaints cause subsequent exclusion from the trade. Furthermore self-reported knee complaints and status of employment through a 10-year follow-up period were analysed.

## Methods

**Figure 1:**
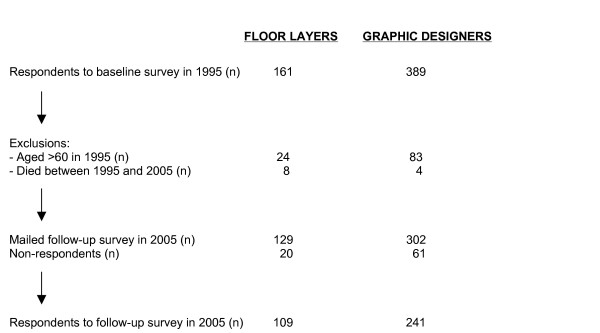
Diagram showing the selection of the final study population.

The study is a follow-up of a cohort of 129 floor layers and 302 graphic designers that in 1994/95 responded to a questionnaire survey at an age less than 60 years and who were alive in 2005 (Figure 1) [4,9]. Graphic designers were included as references due to the fact that their work does not include any knee demanding work tasks. Non-respondents at the follow-up survey constituted 20 floor layers and 61 graphic designers, whereas 8 floor layers and 4 graphic designers had deceased (Figure 1). This leaves 109 and 241 respondents among floor layers and graphic designers, respectively. Among those 81 floor layers and 173 graphic designers were presently at work in their trades in year 1995. All participants were men, living in the area of Copenhagen.

A postal questionnaire was mailed to the cohort. Prior, the Danish Data Protection Agency and the regional ethics committee approved the investigation. In lack of response the questionnaire was filled in by a structured telephone interview, if willing to participate. The method and the applied questionnaire was the same as used in the 1995 study and questions about musculoskeletal complaints were consistent with the Nordic Musculoskeletal Questionnaire [14]. The questionnaire asked about present and previous employment and the history of any knee complaints or knee traumas. In regard to employment we asked about their seniority (years of employment in the trade) and to describe their work tasks. If no longer engaged in the trade they were asked which year they stopped and if this were due to illness, unemployment, early retirement, old-age pension, disablement pension, rehabilitation or change of occupation. In the questionnaire, knee complaints were defined as ache, pain or nuisance. In case of knee complaints they reported if complaints had been present during the past 12 months or daily or more than 30 days during the past 12 months.

Data was coded in the software package EpiData and statistical analysis conducted with Stata [15,16]. Using Cox proportional hazard regression, we analysed the probability of surviving in the trade with knee complaints (>30 days during the past 12 months at baseline) during the 10-year follow-up from 1994/95 to 2005. Furthermore, logistic regression was used to analyse patterns of self-reported knee complaints relative to employment in the trade in year 2005. All analysis was conducted on floor layers (N = 81) and graphic designers (N = 173) presently working in their trades in year 1995. Models were adjusted for effects of body mass index (BMI) and earlier knee injuries. Seniority showed a stronger association than age and was used in the model. The continuous covariates, BMI and seniority, were categorized into three BMI groups (< 25, 25–29 & ≥ 30 kg/m^2^) and two seniority groups (≤ 20 & > 21 years).

## Results

Responses to the mailed follow-up survey were received from 109 floor layers and 241 graphic designers (Figure 1). Among those 36% and 31% had answered the questionnaire by a structured interview. Graphic designers were slightly older and had a higher seniority than floor layers but in respect to body mass index the two groups were comparable (Table 1).

**Table 1:** Demographic and anthropometric characteristics of questionnaire respondents in 2005

	**Floor layers (N = 109)**				**Graphic designers (N = 241)**			
	**1995**		**2005**		**1995 **		**2005**	
	
	Mean	Range	Mean	Range	Mean	Range	Mean	Range
Age (years)	43.5	26–60	53.5	36–70	48.6	30–60	58.6	40–70
Seniority * (years)	22.0	2–40	29.4	3–49	27.6	1–45	34.9	2–54
BMI^‡^(kg/m^2^)	25.5	19.6–40.1	26.7	20.0–51.1	25.0	16.8–37.3	26.1	16.6–42.0

Employment in the trade	n (%)		n (%)		n (%)		n (%)	

Presently at work	**81 **(74.3%)		56 (51.4%)		**173 **(71.8%)		75 (31.1%)	
Presently off work	28 (25.7%)		53 (48.6%)		68 (28.2%)		166 (68.9%)	
*- Change of occupation*	*18 (16.5%)*		*27 (24.8%)*		*37 (15.3%)*		*69 (28.7%)*	
*- Early retirement*	*2 (1.9%)*		*9 (8.3%)*		*27 (11.2%)*		*68 (28.2%)*	
*- Old-age pension*	*-*		*5 (4.6%)*		*-*		*19 (7.9%)*	
*- Disability pension*	*7 (6.4%)*		*8 (7.3%)*		*4 (1.7%)*		*8 (3.3%)*	
*- Rehabilitated*	*-*		*1 (0.9%)*		*-*		*-*	
*- Long illness*	*1 (0.9%)*		*3 (2.7%)*		*-*		*2 (0.8%)*	

*Duration of employment in the trade

^‡^BMI, body mass index

### Employment status in 2005 compared to 1995

Of the respondents to the follow-up survey, 81 (74%) floor layers and 173 (72%) graphic designers had been working in their trade at the time of baseline survey in 1995 (and hence "at risk" of leaving the trade during follow-up). At the time of the follow-up survey in 2005, only 56 (51%) floor layers and 75 (31%) graphic designers were still working in their respective trades (Table 1).

### Knee complaints & survival in the trade 1995–2005

**Figure 2:**
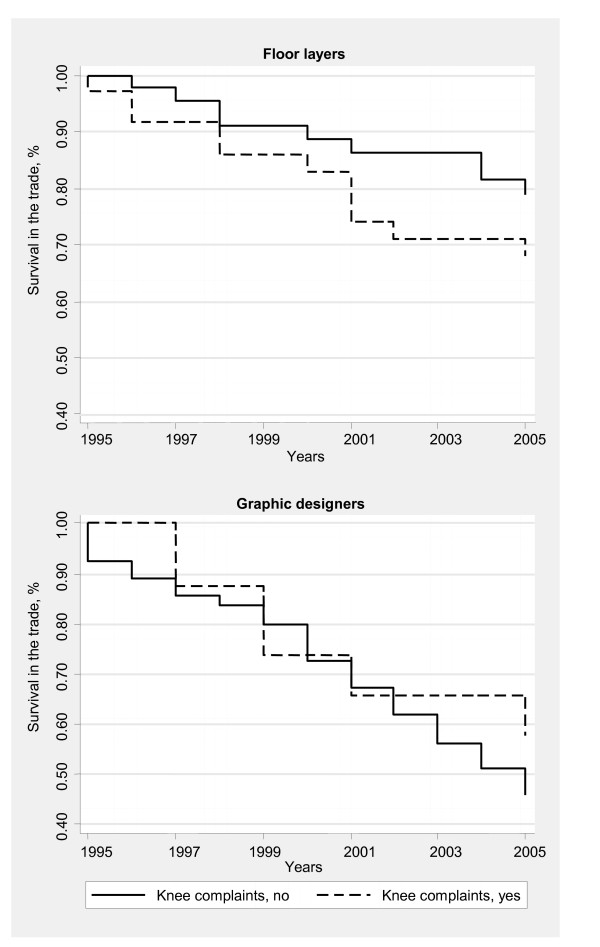
Kaplan-Meier survival curve during a ten years follow-up period (1995–2005) according to self-reported knee complaints (> 30 days during the past 12 months) in 1995 among floor layers (N = 81) & graphic designers (N = 173) presently at work in the trade at baseline (1995).

Analysis among floor layers (N = 81) and graphic designers (N = 173) "at risk" of leaving the trade during follow-up, indicated that the probability of surviving in the trade was higher among floor layers without knee complaints (> 30 days during the past 12 months) in 1995 than among those with knee complaints, although the difference did not reach statistical significance (Figure 2). Among graphic designers proportions were almost identical in the two groups. The Hazard Ratios adjusted for seniority, BMI and earlier knee injuries were 1.4, 95% CI = 0.6–3.5 for floor layers and 0.8, 95% CI = 0.4–1.9 for graphic designers.

### Knee complaints & status of employment in 2005

In regard to status of employment there had been a high employment turnover through the follow-up period. A high percentage was engaged in other trades or had been retired prematurely. Among those who reported change of occupation in the period 1995–2005, 18 floor layers had left the trade before 1995 and 9 in the period 1995–2005. Among graphic designers 37 had changed before 1995 and 32 in the period 1995–2005.

Table 2 depicts the odds risk ratio of knee complaints among 81 floor layers compared to 173 graphic designers that were presently working in their trade in 1994/95. Knee complaints, more than 30 days during the past 12 months, among graphic designers presently at work in the trade, were twice as frequent (20.7%) in 2005 compared with results at baseline (1995) (10.7%). On the contrary, knee complaints > 30 days during the past 12 months, were more frequent at baseline (41.1%) compared with results at follow-up (26.3%) among floor layers presently at work in the trade as well as for floor layers pensioned (50.0% compared to 29.0%). Among those who had changed occupation there were minor differences in the frequency of these knee complaints. Floor layers had significant more knee complaints than graphic designers, if they were no longer engaged in the trade. Among floor layers presently at work in the trade, there were no significant differences for knee complaints > 30 days during the past 12 months compared with graphic designers (OR = 1.4, 95% CI = 0.6–3.3).

**Table 2:** Odds risk ratio of self-reported knee complaints in 1994/95 and 2005 among 81 floor layers presently working in the trade in 1994/95 relative to graphic designers

**Knee complaints**	**Employment in the trade in year 2005**	**Knee complaints 1995 **						**Knee complaints 2005**				
		
		**N **	**n (%) **	**OR_crude_**	**95% CI **^‡^	**OR_adj_^†^**	**95% CI^‡^**	**n (%) **	**OR_crude_**	**95% CI^‡^**	**OR_adj_^†^**	**95% CI **^‡^
	Presently at work											
	- Floor layers	56	39 (69.6%)	8.5	3.8–18.7	9.2	3.8–22.2	32 (56.1%)	2.2	1.1–4.5	2.7	1.2–5.8
	- Graphic designers	75	16 (21.3%)	1	-	1	-	28 (36.8%)	1	-	1	-

During the past 12 months	Presently off work *											
	- Floor layers	16	10 (62.5%)	5.1	1.6–16.3	7.7	2.1–28.9	7 (43.8%)	1.9	0.6–5.9	2.7	0.8–8.9
	- Graphic designers	66	16 (24.2%)	1	-	1	-	19 (28.8%)	1	-	1	-

	Change of occupation											
	- Floor layers	9	8 (88.9%)	27.4	2.9–258.4	26.9	2.7–265.0	7 (70.0%)	7.7	1.4–43.9	11.7	1.1–124.8
	- Graphic designers	32	7 (21.9%)	1	-	1	-	10 (31.3%)	1	-	1	-

	Presently at work											
	- Floor layers	56	23 (41.1%)	5.8	2.3–14.2	5.5	2.2–13.9	15 (26.3%)	1.3	0.6–.2.9	1.4	0.6–3.3
	- Graphic designers	75	8 (10.7%)	1	-	1	-	17 (20.7%)	1	-	1	-

Daily or > 30 days during the past 12 months	Presently off work *											
	- Floor layers	16	8 (50.0%)	7.1	2.1–24.3	9.1	2.4–34.4	5 (29.0%)	2.6	0.7–8.9	3.8	1.0–15.0
	- Graphic designers	66	8 (12.1%)	1	-	1	-	10 (16.7%)	1	-	1	-

	Change of occupation											
	- Floor layers	9	5 (55.6%)	11.7	2.0–68.8	15.3	2.1–113.4	5 (50.0%)	8.8	1.6–47.0	13.8	1.7–115.1
	- Graphic designers	32	3 (9.4%)	1	-	1	-	4 (12.5%)	1	-	1	-

* Off work due to early retirement, old – age pension, disability pension, rehabilitation or long illness

^† ^OR, odds risk ratio adjusted for seniority, body mass index & earlier knee injuries

^‡ ^CI, confidence interval

## Discussion

In the present study we found a positive, although non-significant trend, between knee complaints and premature exclusion from a knee demanding occupation. We did not follow floor layers from their first year in the trade but re-examined a cross-sectional sample including floor layers that had worked in the trade for many years. A bias towards the null because of the well-described healthy worker effect is very likely. The fact that 28 floor layers already had left their trade in year 1994/95 is consistent with this assumption. Questionnaire reports in 1994/95 from these individuals showed that 20% of the floor layers and 4% of graphic designers had been re-educated in another occupation due to knee troubles. Exclusion from the trade may occur at a lower seniority among many floor layers compared to participants included in this study (seniority (average) in 2005 29.4–34.9 years). Therefore, having knee complaints it may not be easy to survive in a knee demanding trade. In earlier studies physical work strains such as lifting and uncomfortable work postures has been found to be associated with early retirement, which support the results from the current study [17,18].

As demonstrated in this follow-up survey, floor layers have a high frequency of knee complaints compared with workers without knee demanding work activities in their work tasks. Earlier studies among floor layers have shown similar results with high frequencies of musculoskeletal complaints, especially knee complaints [8-12]. However, in spite of the increasing age of the cohort the frequency of self-reported knee complaints was in general lower among floor layers presently at work in the trade in year 2005 compared with the frequency at baseline (1995), while the opposite tendency was seen among graphic designers. This could indicate a healthy worker effect among floor layers and moreover, that graphic designers may have an increased possibility to survive in the trade with knee troubles [19]. Knee complaints may therefore be a risk factor of premature resignation from a trade, which involves knee demands and/or other physical work demands and a surviving factor in trades with few physical demands (graphic designers).

In order to prevent occupational musculoskeletal disorders, wearing-down and exclusion from the trade, major efforts have been made during the last ten years to reduce the daily amount of knee straining work activities among Danish floor layers. Among other things, innovations such as the development of tools that can be used in the upright working position have been enhanced and to some extent implemented in the floor layers work tasks [20]. Among those using the equipment, results have shown that particularly severe knee complaints can be reduced and furthermore the effect is greater the longer the equipment have been used [21]. In the light of the reduced frequency of knee complaints observed among floor layers presently at work in the trade in year 2005, this may indicate a certain extent of efficient prevention.

Apart from occupational hazards, it is evident that other aspects also affect the risk of exclusion or early retirement from the labour marked. Previous studies have ruled out the importance of other determinants that may contribute to the disability risk, such as individual factors (physical and psychological health status) and socio-economic conditions [17,22].

During the period 1994/95–2005 the graphic subject area has been affected by structural changes, which have caused a high degree of unemployment and forced many graphic designers into other jobs and trades. Among graphic designers, these structural changes had a strong implication on future exclusion from the trade.

The overall response rate to the questionnaire was acceptable but there may be a risk of interview bias among those contacted by phone. To minimize this, a trained interviewer made all the interviews using a structured guide closely corresponding to the postal questionnaire. Using self-reported data there may additionally be a risk of information bias, e.g. subjects with a previous history of knee problems may have a tendency to respond more readily and accurate than those without [2]. By comparing the answers from questionnaire and interview respondents there was a slightly lower frequency of knee complaints among those who were contacted by phone (but not significant), why the influence of interview bias is considered to be negligible.

In regard to potential confounders we have adjusted the results for some important determinants of self-reported knee complaints, such as seniority, earlier knee injuries and weight (BMI).

We have managed to obtain information's from the majority of the baseline cohort despite a very high occupational mobility among study subjects in both trades. However, a limitation of the present study is the small size of the study population, which may affect the precision of the results and confidence intervals. Subjects aged > 70 years in 2005 were excluded from the follow-up study. In Denmark workers can retire at the age of 60 years. Even if possible to stay at work after the age of 60 years it happens very seldom among construction workers and the risk of missing information owing to these reasons may be small.

## Conclusion

This follow-up survey confirms a positive, although non-significant trend, between knee complaints and premature exclusion from a knee demanding occupation. The study also indicates a healthy worker effect among floor layers and a survivor effect among graphic designers.

## Competing interests

The author(s) declare that they have no competing interests.

## Authors' contributions

SR participated in the design of the study, in the acquisition of data, performed the statistical analysis and participated in the interpretation of the data. LKJ and JPB participated in the design of the study and in the analysis and interpretation of data. All authors have been involved in drafting the manuscript and approved the final manuscript.

## Pre-publication history

The pre-publication history for this paper can be accessed here:



## References

[B1] Arndt V, Rothenbacher D, Daniel U, Zschenderlein B, Schuberth S, Brenner H (2005). Construction work and risk of occupational disability: a ten years follow up of 14 474 male workers. Occup Environ Med.

[B2] Baker P, Reading I, Cooper C, Coggon D (2003). Knee disorders in the general population and their relation to occupation. Occup Environ Med.

[B3] Enderlein G, Kasch J (1989). Modeling of dose-response relations in exposure-related changes of the locomotor system. [Modellierung von Dosis-wirkungsbeziehungen für expositionsabhängige veränderungen am bewegungsapparat] [in German]. Z Gesamte Hyg.

[B4] Jensen LK, Eenberg W (1996). Occupation as a risk factor for knee disorders. Scand J Work Environ Health.

[B5] O'Reilly SC, Muir KR, Doherty M (2000). Occupation and knee pain: a community study. Osteoarthritis Cartilage.

[B6] Coggon D, Croft P, Kellingray S, Barrett D, McLaren M, Cooper C (2000). Occupational physical activities and osteoarthritis of the knee. Arthritis Rheum.

[B7] Hunter DJ, March L, Sambrook PN (2002). Knee osteoarthritis: The influence of environmental factors. Clin Exp Rheumatol.

[B8] Ekström H, Engholm G, Nyqvist B, Wallenquist A (1983). Knee disorders as a occupational problem. [Knäbesvär som arbetsmedicinskt problem Stockholm] [in Swedish]. Bygghälsans Forskningsstiftelse.

[B9] Jensen LK, Mikkelsen S, Loft IP, Eenberg W (2000). Work-related knee disorders in floor layers and carpenters. J Occup Environ Med.

[B10] Kivimäki J, Riihimäki H, Hänninen K (1992). Knee disorders in carpet and floor layers and painters. Scand J Work Environ Health.

[B11] Thun M, Tanaka S, Smith AB, Halperin WE, Lee ST, Luggen ME, Hess EV (1987). Morbidity from repetitive knee trauma in carpet and floor layers. Br J Ind Med.

[B12] Myllymäki T, Tikkakoski T, Typpö T, Kivimäki J, Suramo I (1993). Carpet-layer's knee. An ultrasonographic study. Acta Radiol.

[B13] Brenner H, Ahern W (2000). Sickness absence and early retirement on health grounds in the construction industry in Ireland. Occup Environ Med.

[B14] Kuorinka I, Jonsson B, Kilbom A, Vinterberg H, Biering-Sørensen F, Andersson G, Jorgensen K (1987). Standardised Nordic questionnaires for the analysis of muskuloskeletal symptoms. App Ergon.

[B15] EpiData Association (2000). EpiData Software V31 Odense, DK.

[B16] StataCorp LP (1996). Statistics/Data Analysis V80 College Station, TX.

[B17] Krause N, Lynch J, Kaplan RD, Goldberg DF, Salonen JT (1997). Predictors of disability retirement. Scand J Work Environ Health.

[B18] Karpansalo M, Manninen P, Lakka TA, Kauhanen J, Rauramaa R, Salonen JT (2002). Physical Workload and Risk of Early Retirement: Prospective Population-Based Study Among Middle-Aged Men. J Occup Environ Med.

[B19] Siebert U, Rothenbacher D, Daniel U, Brenner H (2001). Demonstration of the healthy worker survivor effect in a cohort of workers in the construction industry. Occup Environ Med.

[B20] Jensen LK, Kofoed LB (2002). Musculoskeletal disorders among floor layers: is prevention possible?. Appl Occup Environ Hyg.

[B21] Jensen LK, Friche C (2007). Effects of training to implement new tools and working methods to reduce knee load in floor layers. Appl Ergon.

[B22] Lissau I, Rasmussen NK, Hesse NM, Hesse U (2001). Social differences and health-related exclusion from the labour market in Denmark from 1987 to 1994. Scand J Public Health.

